# Differential susceptibility and maturation of thymocyte subsets during *Salmonella* Typhimurium infection: insights on the roles of glucocorticoids and Interferon-gamma

**DOI:** 10.1038/srep40793

**Published:** 2017-01-16

**Authors:** Shamik Majumdar, Mukta Deobagkar-Lele, Vasista Adiga, Abinaya Raghavan, Nitin Wadhwa, Syed Moiz Ahmed, Supriya Rajendra Rananaware, Subhashish Chakraborty, Omana Joy, Dipankar Nandi

**Affiliations:** 1 Department of Biochemistry, Indian Institute of Science, Bangalore 560012, India; 2 Centre for Infectious Disease Research, Indian Institute of Science, Bangalore 560012, India; 3 Flow Cytometry Facility, Indian Institute of Science, Bangalore 560012, India

## Abstract

The thymus is known to atrophy during infections; however, a systematic study of changes in thymocyte subpopulations has not been performed. This aspect was investigated, using multi-color flow cytometry, during oral infection of mice with *Salmonella* Typhimurium (*S*. Typhimurium). The major highlights are: First, a block in the developmental pathway of CD4^−^CD8^−^ double negative (DN) thymocytes is observed. Second, CD4^+^CD8^+^ double positive (DP) thymocytes, mainly in the DP1 (CD5^lo^CD3^lo^) and DP2 (CD5^hi^CD3^int^), but not DP3 (CD5^int^CD3^hi^), subsets are reduced. Third, single positive (SP) thymocytes are more resistant to depletion but their maturation is delayed, leading to accumulation of CD24^hi^CD3^hi^ SP. Kinetic studies during infection demonstrated differences in sensitivity of thymic subpopulations: Immature single positive (ISP) > DP1, DP2 > DN3, DN4 > DN2 > CD4^+^ > CD8^+^. Upon infection, glucocorticoids (GC), inflammatory cytokines, e.g. Ifnγ, etc are induced, which enhance thymocyte death. Treatment with RU486, the GC receptor antagonist, increases the survival of most thymic subsets during infection. Studies with *Ifnγ*^−/−^ mice demonstrated that endogenous Ifnγ produced during infection enhances the depletion of DN2-DN4 subsets, promotes the accumulation of DP3 and delays the maturation of SP thymocytes. The implications of these observations on host cellular responses during infections are discussed.

T lymphocytes develop in the thymus where they undergo differentiation, selection and maturation. The earliest thymocyte precursors lack cell surface expression of CD4 and CD8 glycoproteins and are referred to as DN thymocytes. Based on CD44 and CD25 expression, DN thymocytes are broadly subdivided into four subsets. The DN1 (CD44^+^CD25^−^) compartment consists of cells that give rise to DN2 (CD44^+^CD25^+^) cells. In turn, DN2 thymocytes downregulate CD44 to form the DN3 (CD44^−^CD25^+^) subset, which is exclusively committed to the T cell lineage. Here, T cell receptor (TCR) β-selection occurs and cells lose CD25 expression, progressing to the DN4 (CD44^−^CD25^−^) cells^1^. The DN4 cells further give rise to ISP thymocytes, which are phenotypically characterized as CD4^−^CD8^+^CD24^hi^CD3^lo^. These cells are Notch-responsive, proliferate rapidly, rearrange the TCRα chain and serve as the precursors of CD4^+^CD8^+^ DP thymocytes^2^,^3^. DP cells are classified into three distinct subsets on the basis of cell surface CD3 and CD5 expression. The DP1 (CD5^lo^CD3^lo^) thymocytes give rise to DP2 (CD5^hi^CD3^int^), which differentiate to CD4^+^ T cells. The DP3 (CD5^int^CD3^hi^) cells arise from DP2 and give rise to CD8^+^ T cells^4^,^5^,^6^. During selection, self-reactive TCR clones are deleted and cell surface CD24 and CD69 are down-modulated before mature SP T cells egress the thymus as recent thymic emigrants^7^,^8^.

An important aspect of the biology of the thymus is its ability to undergo involution, known as thymic atrophy, which occurs naturally during the process of aging^9^,^10^. In addition, the thymus is exquisitely sensitive to starvation, stress, inflammation, infection, etc.^11^,^12^,^13^. Infection by microbes or the presence of microbial components cause thymic atrophy^11^,^14^. A characteristic feature of thymic atrophy is the apoptosis of DP thymocytes^15^,^16^,^17^, although in some cases, other populations are also affected^18^,^19^,^20^. Over a prolonged period of time, thymic atrophy may lead to lower thymic output. Under these conditions, the maintenance of the peripheral T cell pool is dependent upon the expansion of pre-existing T cells, resulting in lower diversity of TCRs. Consequently, there is an increased risk of infection, development of tumours, etc. due to compromised cellular immune responses.

Several factors are responsible for infection-induced thymic atrophy. One such key mediator is the increase in stress-induced GC amounts. Several reports have demonstrated the roles of GCs either by adrenalectomy or administration of RU486, a GC receptor signalling antagonist^15^,^16^,^17^. Another set of players that are important are inflammatory cytokines^14^,^17^,^20^,^21^. Infection with the human immunodeficiency virus - 1 alters the cytokine profiles of infected thymocytes^22^ and the role of tumour necrosis factor alpha (Tnfα) has been shown in several infections^15^,^21^. In addition, cytokines e.g. IL6 and its family members^23^, and other molecules, e.g. reactive oxygen species^24^, signalling molecules^25^, and caspases^26^ play important roles during infection-induced thymic atrophy.

Our laboratory has previously reported that extensive thymic atrophy occurs during *S.* Typhimurium infection^17^. *S.* Typhimurium is a Gram negative bacterium that survives and replicates within intracellular compartments in host cells. It causes gastroenteritis in humans but leads to typhoid-like disease in mice, similar to that caused by *S.* Typhi in humans^27^. There are two main factors that highlight the importance of understanding different aspects of *Salmonella* pathogenesis: first, the rise of multi-drug resistant strains^28^ and, second, the susceptibility of immunocompromised people, e.g. human immunodeficiency virus infected cohorts in Africa, to *Salmonella* infections^29^. Using a mouse model of *S*. Typhimurium-induced thymic atrophy, we have shown that DPs, but not SPs, undergo apoptosis^17^. Infection-induced thymic atrophy is well documented; however, the changes in the thymic subpopulations and the underlying mechanisms constitute a relatively less explored area of research. In this study, we report the effects of infection on different thymocyte subsets using multi-colour flow cytometry. Also, the roles of GC and endogenous Ifnγ were investigated and the implications of this study, in the context of immune consequences during infection-induced thymic atrophy, are discussed.

## Results

### Acute thymic atrophy during *S*. Typhimurium infection leads to loss of DN and DP thymocyte subsets

**Figure 1:**
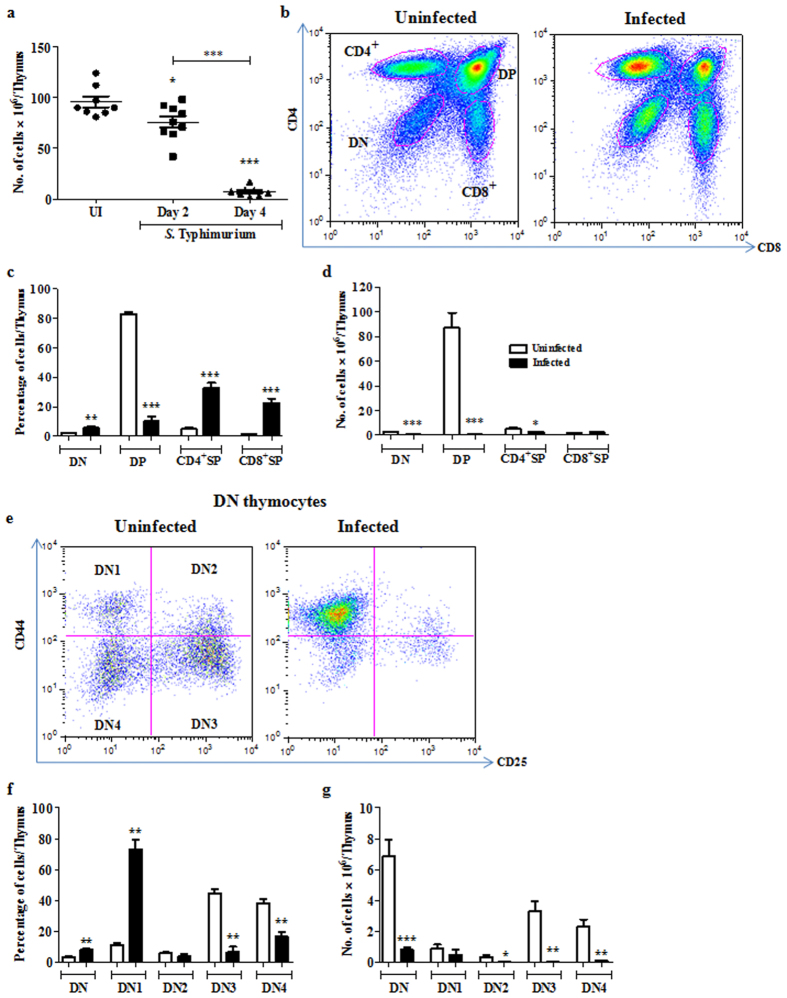
Acute loss of DN and DP thymocytes occurs during *S*. Typhimurium infection-induced thymic atrophy. C57BL/6 mice were orally infected with *S*. Typhimurium and sacrificed on day 4 post infection along with uninfected (UI) controls. (**a**) Viable cell numbers from thymi were quantified by Trypan blue exclusion assay. (**b**) Thymocytes were stained for surface expression of CD4 and CD8 and the (**c**) percentage and (**d**) numbers of the major subsets (DN, DP, CD4^+^ SP, CD8^+^ SP cells) were enumerated. (**e**) CD44 and CD25 profile of the DN population was plotted and (**f**) percentages and the (**g**) number of cells in each DN subset was quantified. Data are shown as mean ± SEM of six to eight mice per group. *p ≤ 0.05, **p ≤ 0.01, and ***p ≤ 0.001, two-tailed Mann-Whitney test.

To study the various subpopulations of the thymus post atrophy, we orally infected C57BL/6 mice with ~10^8^ CFU of *S*. Typhimurium and sacrificed them on day 2 and 4 post infection^17^. As previously reported^17^, the infection load increased from day 2 to day 4 in Peyer’s patches, liver and spleen. In the MLN, the reduction in cellularity was about 2 fold on day 4. In contrast, 8–10 fold fewer viable thymocytes were recovered on day 4 post infection, as compared to uninfected or day 2 infected mice, indicating massive loss of thymocytes during later stages of infection (Fig. 1a). Also, thymi from day 4 infected mice were visibly smaller than those from uninfected control mice, demonstrating infection-induced thymic atrophy (Fig. S1a).

To phenotypically characterize the major cell populations in the thymus, cells were stained for surface expression of CD4 and CD8 (Fig. 1b). DP thymocytes which constitute ~80% of total thymocytes were significantly reduced, resulting in an apparent increase in the percentages of DN and SP populations upon infection (Fig. 1c). However, the total cellularity of the thymus also decreased by 8–10 fold (Fig. 1a). Since the increase in the percentage of a population does not always correlate with its absolute numbers, the data in this and subsequent figures are plotted as absolute cell numbers. Overall, a significant depletion of DN and DP thymocytes and a moderate reduction of CD4^+^, but not CD8^+^, SP thymocytes was observed (Fig. 1d).

### Infection leads to extensive depletion of DN2, DN3 and DN4 cells in the DN thymocyte subset

Next, we performed a systematic analysis of individual thymocyte subsets belonging to the major thymocyte populations viz., DN, DP and SP subsets. To investigate the changes in the earliest thymocyte precursors gated as DN population lacking CD4 and CD8 (Fig. 1b), we analyzed the CD44 and CD25 expression profiles upon infection (Fig. 1e). Post atrophy, the percentage of DN1 cells increased whereas DN3 and DN4 cells reduced (Fig. 1f). The DN2, DN3 and DN4 cell numbers were significantly reduced in contrast to the DN1 subset, which remained largely unchanged (Fig. 1g).

### DP3 cells are more resistant to depletion compared to DP1 and DP2 subsets during infection

**Figure 2:**
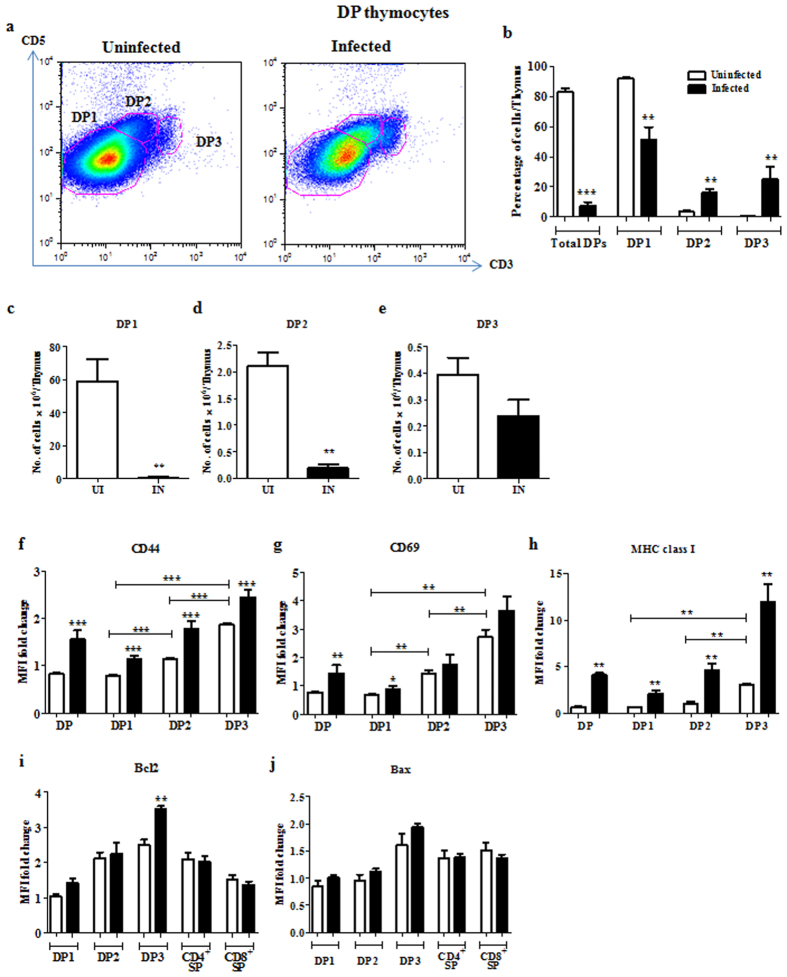
Immature DP1 and DP2, but not DP3, thymocytes are greatly depleted upon *S.* Typhimurium infection. Thymocytes from uninfected (UI) and infected (IN) mice were isolated and stained for surface expression of CD4, CD8, CD3, CD5, CD44, CD69 and MHC class I. (**a**) Representative plots for expression of CD5 and CD3 in the CD4^+^ CD8^+^ population are shown. (**b**) The percentages and total number of cells in (**c**) DP1, (**d**) DP2 and (**e**) DP3 subsets within the CD4^+^ CD8^+^ population was quantified. The fold change in MFI of cell surface (**f**) CD44, (**g**) CD69, (**h**) MHC class I and intracellular (**i**) Bcl2 and (**j**) Bax in the DP and SP subpopulations was quantified. Data are shown as mean ± SEM of four to eight mice per group. *p ≤ 0.05, **p ≤ 0.01, and ***p ≤ 0.001, two-tailed Mann-Whitney test.

To investigate the susceptibility of DP subsets to depletion, we analyzed CD5 and CD3 profiles of DPs from uninfected and day 4 infected mice (Fig. 2a). In earlier studies, CD5 and αβTCR were used for characterisation of DP subsets^4^,^5^. As TCRs are co-expressed with the CD3 complex^30^,^31^, we compared the CD5 versus αβTCR and CD5 versus CD3 plots, using different fluorescent conjugates (Fig. S2). Since DP subsets were best resolved using anti-CD5-FITC and anti-CD3-V450, we used this combination for our studies. The DP cells were classified on the basis of CD5 and CD3 expression as DP1 (CD5^lo^CD3^lo^), DP2 (CD5^hi^CD3^int^) or DP3 (CD5^int^CD3^lo^) (Fig. S3). The percentage of DP1 reduced with infection whereas the percentages of DP2 and DP3 increased (Fig. 2b). The DP1 and DP2 cell numbers were significantly reduced, whereas DP3 cells were comparatively more resistant to depletion during infection (Fig. 2c–e). We also monitored the expression of the activation/maturation markers, CD44 (Fig. 2f), CD69 (Fig. 2g) and MHC class I (Fig. 2h) in the three DP subsets. As the DP cells matured, the expression of CD44 and CD69 increased from DP1 to DP2 to DP3, while a significant increase in the expression of MHC class I was seen in DP3 (Fig. 2f–h). Importantly, upon infection the residual DP cells in the three subsets expressed higher amounts of CD44 and MHC class I compared to the uninfected controls, indicating survival of the more activated and/or mature DP cells (Fig. 2f,h).

To understand factors that may be important in the resistance of DP3 cells to depletion, we assessed the expression of pro- and anti-apoptotic molecules, Bax and Bcl2, respectively. The surviving DP3 cells expressed elevated amounts of intracellular Bcl2 compared to DP1 and DP2 subsets (Fig. 2i). On the other hand, the intracellular Bax amounts were not significantly altered among the DP subsets upon infection (Fig. 2j).

### CD8^+^ ISP thymocytes are completely depleted during infection with an increase in CD24^hi^CD3^hi^ SP cells

**Figure 3:**
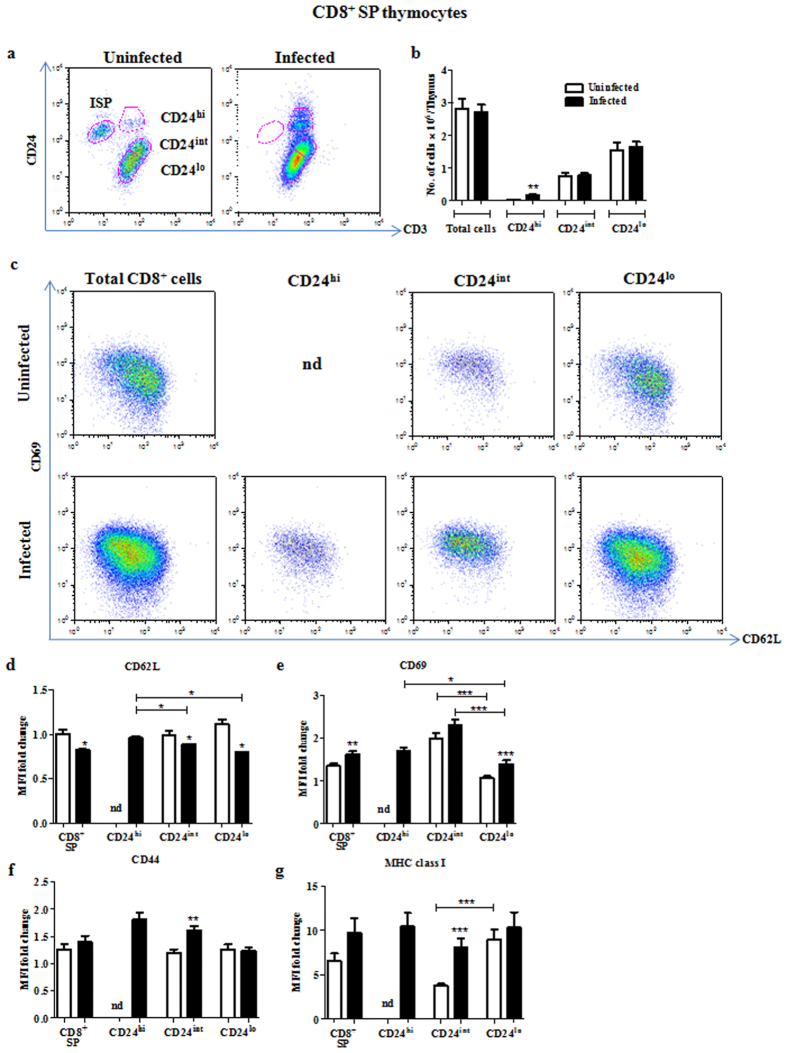
Complete loss of ISP cells, i.e. CD24^hi^CD3^lo^CD8^+^, and increase in less mature CD24^hi^CD3^hi^CD8^+^ thymocytes occurs upon infection. Thymocytes from uninfected and day 4 infected mice were isolated and stained for surface expression of CD4, CD8, CD3, CD24 or CD44 or MHC class I or CD69 and CD62L. (**a**) CD8^+^ SP cells were gated as ISP, CD24^hi^, CD24^int^ or CD24^lo^ on the basis of surface expression of CD24 and CD3. (**b**) The number of cells in the CD24^hi^, CD24^int^ or CD24^lo^ subsets was quantified. (**c**) The CD69 and CD62L density profiles of these subsets are depicted. Fold change in the MFI of (**d**) CD62L, (**e**) CD69, (**f**) CD44 and (**g**) MHC class I were calculated for the different CD8^+^ SP subpopulations. Data are shown as mean ± SEM of four to eight mice per group. *p ≤ 0.05, **p ≤ 0.01, and ***p ≤ 0.001, two-tailed Mann-Whitney test.

Cell surface markers such as CD3, CD44, CD69, MHC class I, etc. are upregulated while others such as CD24 are down-regulated as DP cells mature into SP thymocytes^32^. Initially, the expression of CD24 and CD3 was analyzed on CD8^+^ SP cells (Fig. 3a). The ISP cells^2^ (CD24^hi^CD3^lo^) were entirely absent in day 4 infected mice displaying acute atrophy (Fig. 3a). In addition, an increase in the number of CD24^hi^CD3^hi^ thymocytes, which are the most immature of the CD8^+^ SP stage, was observed (Fig. 3a,b). The CD8^+^CD3^hi^ SP cells were gated as CD24^hi^, CD24^int^ or CD24^lo^ and their maturation status was analyzed using CD69 and CD62L^33^. The CD24^int^ cells from uninfected mice are CD69^hi^CD62L^lo^ and are comparatively less mature. On the other hand, CD24^lo^ cells are more mature, i.e. CD69^lo^CD62L^hi^. Upon infection, there is a shift towards a less mature and/or more activated phenotype, i.e. CD69^hi^CD62L^lo^ (Fig. 3c–e). CD44 was expressed equally on CD24^int^ and CD24^lo^ cells in controls, while it was significantly upregulated upon infection on the CD24^int^ and CD24^hi^ cells (Fig. 3f). Also, in uninfected mice, CD24^int^ cells were MHC class I^lo^, while CD24^lo^ cells were MHC class I^hi^ (Fig. 3g). However with infection, there was upregulation of MHC class I in CD24^int^ cells, resulting in both CD24^int^ and CD24^lo^ cells having comparable levels of the marker (Fig. 3g).

**Figure 4:**
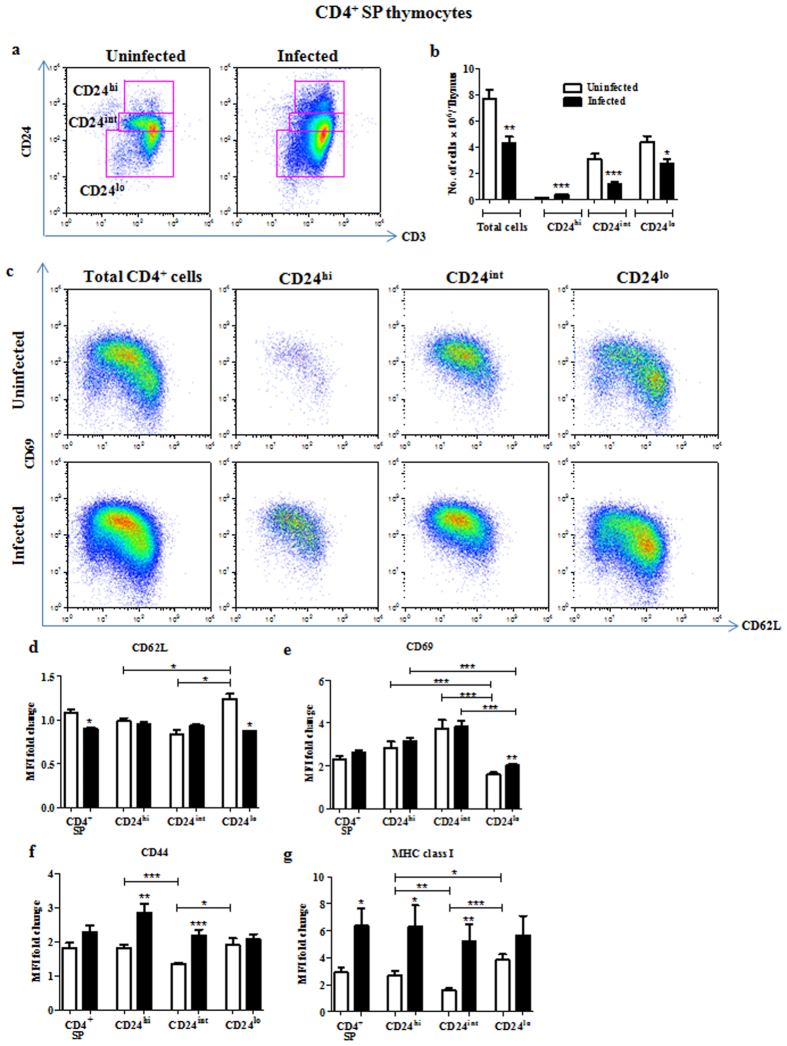
Accumulation of less mature CD24^hi^CD3^hi^CD4^+^ thymocytes is observed during *S*. Typhimurium infection. On day 4 post infection, thymocytes from uninfected and infected mice were stained for surface expression of CD4, CD8, CD3, CD24 or CD44 or MHC class I or CD69 and CD62L. (**a**) CD4^+^ SP cells were gated as CD24^hi^, CD24^int^ and CD24^lo^ on the basis of the expression of CD24 and CD3. (**b**) The number of cells in these subsets was quantified and (**c**) CD69 and CD62L expression are shown. Fold change in MFI of (**d**) CD62L, (**e**) CD69, (**f**) CD44 and (**g**) MHC class I were calculated for the different CD4^+^ SP subpopulations. Data are shown as mean ± SEM of four to eight mice per group. *p ≤ 0.05, **p ≤ 0.01, and ***p ≤ 0.001, two-tailed Mann-Whitney test.

Similar studies were performed in the CD4^+^ SP compartment. Accumulation of CD24^hi^ cells was also observed upon infection (Fig. 4a,b). Similar to the corresponding populations in CD8^+^ SP compartment, CD24^hi^ and CD24^int^ cells were found to be phenotypically less mature i.e., CD69^hi^CD62L^lo^ (Fig. 4c–e). Upon infection, these as well as the CD24^lo^ cells displayed a less mature and/or a more activated phenotype, i.e. CD69^hi^CD62L^lo^ (Fig. 4c–e). Staining for the other maturation markers revealed CD24^int^ cells from uninfected mice to have a phenotype, CD69^hi^MHC class I^lo^, while CD24^lo^ cells showed the phenotype of CD69^lo^MHC class I^hi^ (Fig. 4e,g). Importantly, upon infection, CD44 and MHC class I expression were upregulated in CD24^hi^ and CD24^int^ cells (Fig. 4f,g).

### All thymocyte subsets are not equally susceptible to depletion during infection

**Figure 5:**
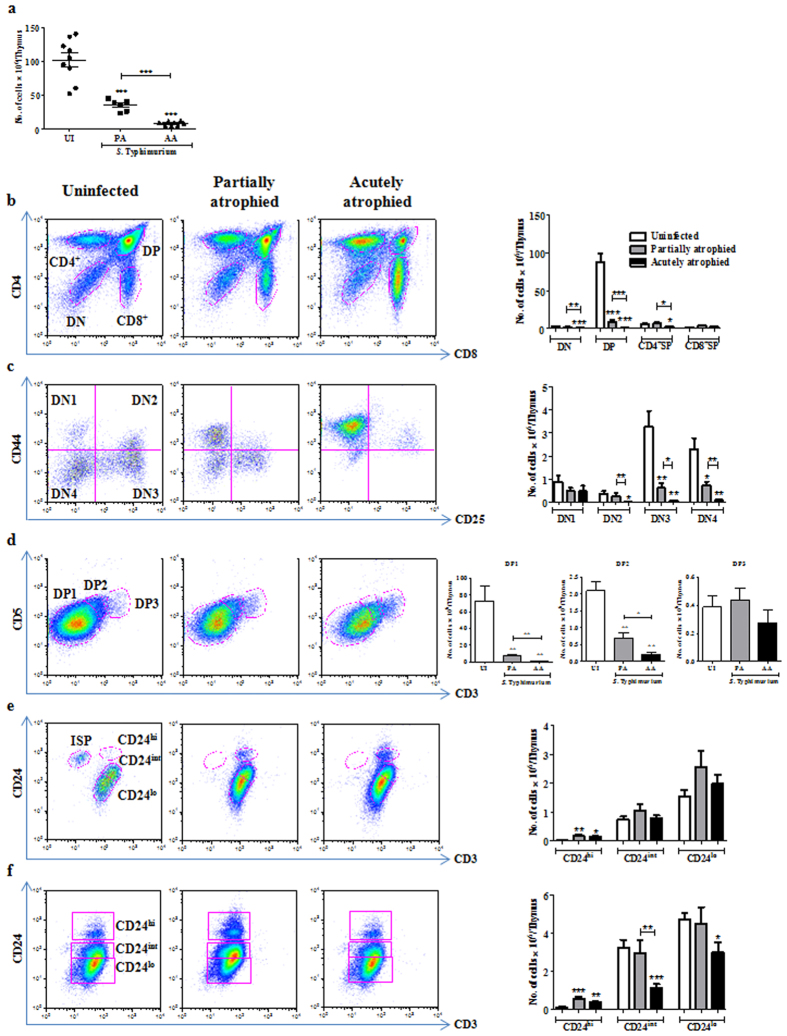
Differential susceptibility of thymocyte subsets is seen upon partial and acute thymic atrophy. Thymocytes from uninfected (UI) and infected mice with partial (PA, day 3) or acute (AA, day 4) thymic atrophy were isolated and (**a**) number of live cells was quantified. Thymocytes from each group were analyzed to quantify the cell numbers in the following populations: (**b**) total DN, DP, CD4^+^ SP, CD8^+^ SP (**c**) DN1, DN2, DN3, DN4 (**d**) DP1, DP2, DP3 (**e**) CD8^+^ SP and (**f**) CD4^+^ SP subsets. Data are shown as mean ± SEM of five to eight mice per group. *p ≤ 0.05, **p ≤ 0.01, and ***p ≤ 0.001, two-tailed Mann-Whitney test.

Next, the degree of susceptibility of individual thymocyte subpopulations was examined. Thymic subsets were studied in three distinct groups of mice: (a) thymi obtained from uninfected (UI) control mice (>40 million thymocytes/organ), (b) partially atrophied (PA) thymi from day 3 infected mice which had 2–4 fold reduction in thymocyte numbers (20–40 million thymocytes/organ) and (c) acutely atrophied (AA) thymi obtained from day 4 infected mice with ~8–10 fold depletion in cellularity (<20 million thymocytes/organ) (Fig. 5a). The bacterial CFU load in Peyer’s patches, liver and spleen was similar in days 3 and 4 post infection (Fig. S4a–c) and no significant difference was found in serum Ifnγ amounts (Fig. S4d). However, the serum Tnfα, Il6 and cortisol amounts were higher in the day 4 infected mice compared to day 3 infected mice (Fig. S4e–g).

Thymocyte from day 3 and day 4 infected mice and uninfected controls were analyzed for surface expression of a wide range of markers (Fig. 5). The DP cell populations were reduced significantly by day 3 and were further depleted by day 4 of infection. Also, CD4^+^ SP cell numbers were comparable to uninfected controls by day 3 and were partially depleted only by day 4 of infection (Fig. 5b). In partially atrophied thymi, we observed a considerable loss of DN3 and DN4 cells on day 3, while on day 4 of infection, the DN2 cells were also reduced. Hence, within the DN population, the DN3 and DN4 cells are more susceptible to depletion by day 3 and are entirely lost by day 4. In contrast, DN2 cells are depleted significantly only by day 4 of infection, whereas the DN1 cells are largely unaffected (Fig. 5c). To summarize, the DN1 cells were the most resistant to depletion, followed by DN2, DN3 and DN4 cells. In the CD8^+^ SP compartment, the ISP cells were completely depleted in the partially atrophied thymi by day 3 (Fig. 5e), possibly resulting in fewer available precursors to DP thymocytes.

In the DP compartment, there was a steep reduction of DP1 and a substantial drop in DP2 cells by day 3 of infection, with no alteration in the DP3 cell population. By day 4 of infection, the DP1 and DP2 cell numbers were further depleted with no significant effect on DP3 cells (Fig. 5d). Among the SP cells, a significant build-up of the less mature CD24^hi^CD3^hi^ cells was observed as early as day 3 of infection with no further increase by day 4 (Fig. 5e,f). Additionally the CD24^int−lo^CD3^hi^CD4^+^ SP cells were also depleted by day 4 (Fig. 5f). Therefore, ISP cells were the most susceptible to depletion followed by DP1 and DP2 cells, DN3, DN4 and DN2 cells and then the CD24^int^ and CD24^lo^ CD4^+^ SP cells.

### Endogenous GC and Ifnγ have an additive role in the depletion of thymocyte subpopulations during infection-induced thymic atrophy

Initially, the effects of RU486 alone on thymocyte subsets in C57BL/6 mice were studied by injecting with vehicle or RU486 alone. The total thymocyte numbers did not vary greatly among the two groups of mice. Importantly, in mice treated with RU486, no profound changes were observed in DN, DP and SP subsets (Fig. S6). Next, the effects of GC and Ifnγ on thymic subpopulations were addressed. As previously shown^17^, administration of the GC receptor antagonist RU486 did not have any major effects on the infection burden, cortisol and the pro-inflammatory cytokine amounts in the sera. Also, higher CFU were recovered from liver and spleen in *Ifnγ*^−/−^ mice compared to BL/6 mice on day 4 post infection^17^. Upon infection, a modest decrease in Tnfα amounts was observed in *Ifnγ*^−/−^ mice while serum cortisol amounts were comparable to those of infected BL/6 mice^17^.

**Figure 6:**
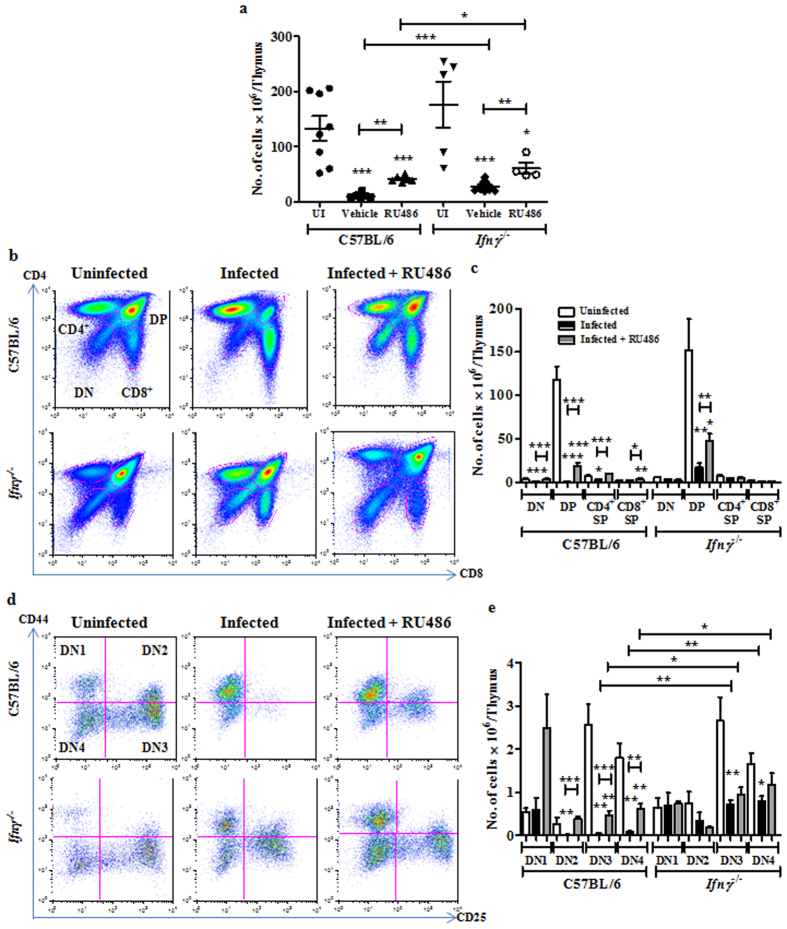
Glucocorticoid signalling and Ifnγ together contribute to the depletion of thymocyte subsets during infection. Post *S*. Typhimurium infection, C57BL/6 and *Ifnγ*^−/−^ mice were administered vehicle or RU486 (20 mg/kg) and sacrificed on day 4 along with uninfected controls (UI). (**a**) The total number of live cells in thymi from each group was quantified. Thymocyte subsets were analyzed for quantification of (**b**,**c**) total DN, DP, CD4^+^ SP, CD8^+^ SP subsets and (**d**,**e**) DN1, DN2, DN3 and DN4 cell numbers. Data are shown as mean ± SEM of four to nine mice per group. *p ≤ 0.05, **p ≤ 0.01, and ***p ≤ 0.001, two-tailed Mann-Whitney test.

A modest but significant rescue in the total number of thymocytes post 4 days of infection was observed in *Ifnγ*^−/−^ mice, as compared to BL/6 mice. Intra-peritoneal administration of RU486 partially rescued the number of thymocytes in BL/6 mice and the extent of rescue was enhanced in *Ifnγ*^−/−^ mice upon infection (Fig. 6a). CD4 and CD8 profiles of thymocytes revealed that the lack of Ifnγ prevented the depletion of DN and CD4^+^ SP thymocytes and partially rescued the loss of DP cells. Administration of RU486 to BL/6 mice rescued the depletion of both DN and CD4^+^ SP cells and to a smaller extent of DP cells. Further increase in the survival of DP cells was observed upon RU486 treatment of *Ifnγ*^−/−^ mice (Fig. 6b,c).

**Figure 7:**
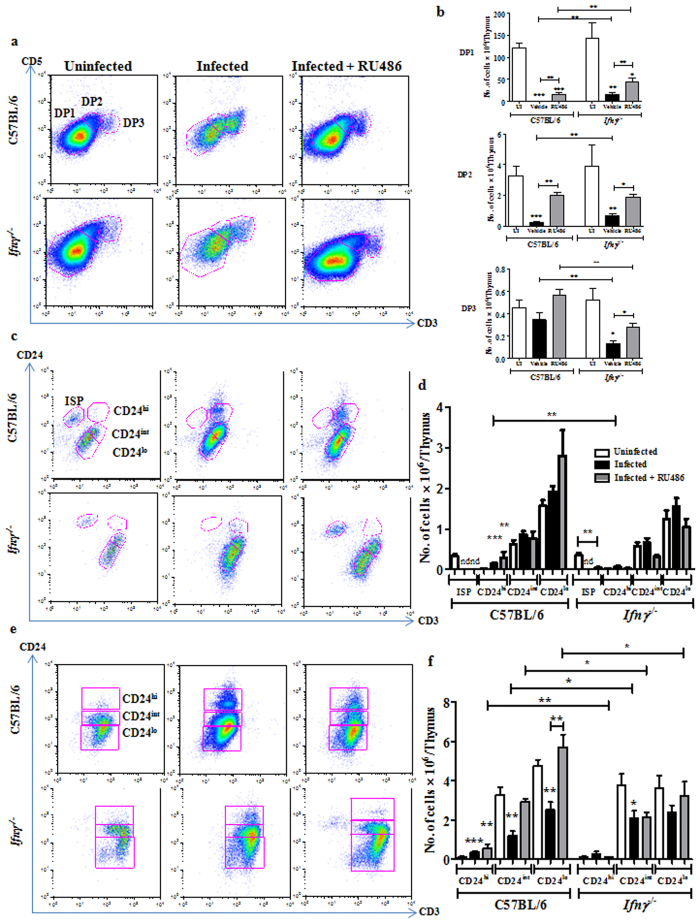
Treatment of Ifnγ lacking mice with RU486 has an additive effect on the survival of different thymic subpopulations during infection. RU486 (20 mg/kg) was administered to C57BL/6 and *Ifnγ*^−/−^ mice post infection. Infected (IN) mice along with the uninfected (UI) controls were sacrificed. Thymocyte subsets (**a**,**b**) DP, (**c**,**d**) CD8^+^ SP and (**e**,**f**) CD4^+^ SP were analyzed and the cell numbers in each subpopulation were quantified. Data are shown as mean ± SEM of four to nine mice per group. *p ≤ 0.05, **p ≤ 0.01, and ***p ≤ 0.001, two-tailed Mann-Whitney test.

Detailed analysis of the DN subset showed that, while the DN1 cells remained unaffected, more DN2-4 cells were recovered from infected *Ifnγ*^−/−^ mice compared to BL/6 (Fig. 6d,e). In BL/6 mice, RU486 completely rescued the loss in DN2 cells whereas the DN3 and DN4 subsets were partially rescued. Interestingly, in some mice, a substantial increase in DN1 thymocytes was observed after RU486 treatment, while in others there was no apparent accumulation. The survival of DN3 and DN4 subsets was higher upon RU486 treatment in *Ifnγ*^−/−^ mice (Fig. 6d,e). ISP thymocytes were not detected in *Ifnγ*^−/−^ mice post infection, indicating that the depletion of this subset was Ifnγ independent. Surprisingly, administration of RU486 post infection led to better survival of ISPs in *Ifnγ*^−/−^ mice, but not BL/6 (Fig. 7c,d).

Analysis of the DP subsets revealed comparable reduction of DP1, DP2 and DP3 subsets in *Ifnγ*^−/−^ mice in contrast to BL/6 infected mice where DP3 cells were more resistant to depletion. RU486 administration increased the survival of DP1 and DP2 cells, which contributed towards the modest increase in the total DP cells in BL/6 mice (Fig. 7a,b). The survival of DP1 and DP2 thymocytes in *Ifnγ*^−/−^ mice was increased by administration of RU486, which is indicative of the additive roles of GC and Ifnγ. Interestingly, RU486 also rescued the DP3 population in *Ifnγ*^−/−^ mice. In the CD4^+^ and CD8^+^ SP populations, fewer CD24^hi^CD3^hi^ thymocytes accumulated in mice lacking Ifnγ. However, the accumulation of less mature CD24^hi^CD3^hi^ cells in both the SP cell subsets was unaffected upon addition of RU486 in BL/6 mice (Fig. 7c,d,e,f).

**Figure 8:**
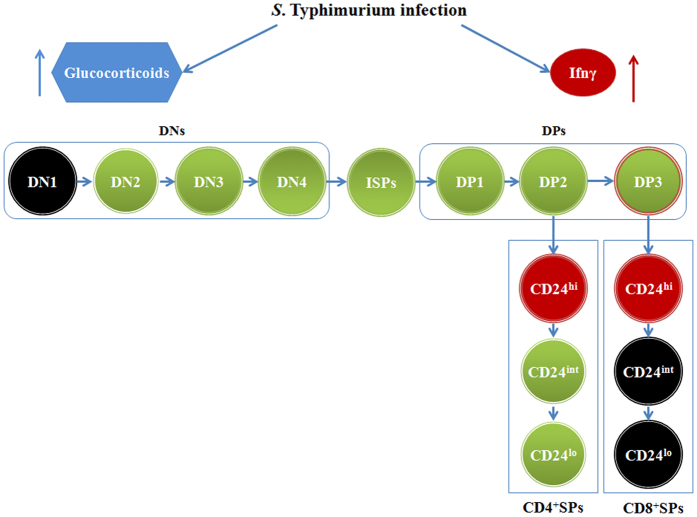
Graphical representation of the effects of glucocorticoids and Ifnγ on thymocyte subpopulations during *S*. Typhimurium infection. During infection, both glucocorticoids and Ifnγ are induced. Glucocorticoids (marked in blue) play a prominent role in depletion of most thymic subsets. Ifnγ (marked in red) is important for the accumulation of CD24^hi^CD4^+^ and CD24^hi^CD8^+^ thymocytes. The combination of glucocorticoids and Ifnγ (marked in green) contributes to the depletion of DN2, DN3, DN4, ISP, DP1, DP2, DP3, CD24^int^CD4^+^ and CD24^lo^CD4^+^ subsets. Notably, DN1, CD24^int^CD8^+^ and CD24^lo^CD8^+^ SP thymocytes are not significantly affected during infection and are represented in black.

The effects of endogenous GC and Ifnγ on thymic subpopulations during infection-induced thymic atrophy are summarized in Fig. 8. Experiments with RU486 clearly showed a major role for GC in the depletion of most thymic subsets affected during infection. On the other hand, elevated Ifnγ amounts during infection are important for the lower accumulation or survival of CD24^hi^CD3^hi^ SP thymocytes. Overall, both cortisol and Ifnγ are important for the survival of most thymic subsets during infection (Fig. 8).

## Discussion

This study has focused on the changes in various thymocyte subsets during an acute and lethal model of oral infection by live *S*. Typhimurium. Time course studies demonstrated that thymocyte subpopulations were differentially susceptible to depletion such that ISP cells were most susceptible to death, followed by DP1, DP2, DN3, DN4, DN2 and to a lesser extent CD4^+^ SP and DP3 cells (Fig. 5). The CD24^int/lo^CD8^+^ SP thymocytes appear to be the most resistant to depletion during infection-induced atrophy. Since GC and Ifnγ are induced upon infection (Fig. S4) and lower the survival of thymocytes^17^, the effects of these molecules on individual thymocyte subsets were analyzed (Figs 6 and 7) and the data is graphically summarized in Fig. 8.

The reduction of DN thymocyte numbers during acute thymic atrophy may be governed by a combination of multiple factors: (1) reduction or complete loss of trafficking of early thymic progenitors from the bone marrow to the thymus^34^ or (2) developmental block at a specific DN stage leading to the accumulation of precursors and reduced pool of thymocytes^35^ or (3) death of all DN subsets^18^,^19^. During chronic infection using live attenuated *Salmonella*, the numbers of all DN subsets reduced^18^. Upon infection of mice with a virulent strain of *Mycobacterium avium*, all DN subsets also reduce^19^. In both these chronic models of infection, thymocytes were analyzed after 21–60 days post infection. On the other hand, our model utilizes a live and virulent bacterial strain and studies were performed by 4 days post infection. Importantly, in our acute infection model, there is differential survival of thymocytes: the immature thymocytes are more sensitive, whereas the more mature thymocytes are more resistant, to death. In this study, fewer DN and no ISP cells were recovered post infection (Figs 1e,g and 3a). The overall reduction of DN thymocytes is likely to be due to a selective depletion of thymocytes from the DN2 stages onwards and not due to a reduction in the influx of bone marrow derived progenitors. This in turn may influence the generation of ISP cells and, along with reduced survival of the ISP cells themselves, may contribute to their complete absence upon infection. It is possible that impairment in cell cycle progression or survival at the DN1 stage may play important roles. Other studies have reported a similar block at the DN1 stage: DN1 cells accumulate during age-associated thymic atrophy^9^ or due to lack of the T cell-specific transcription factor, Tcf-1 in older mice^10^. Further studies are required to understand the factors governing the depletion of DN2-ISP cells in the context of an infection.

The depletion of DP thymocytes has been widely reported during infections by pathogens including bacteria^15^,^17^, viruses^20^,^22^,^24^ and parasites^16^,^21^,^26^. We observed that DP1 and DP2 cells were highly susceptible to depletion whereas the DP3 subset was comparatively resistant during infection (Fig. 2a–e). It has been well established that as DP cells mature they acquire the CD5^hi^CD69^hi^TCR^hi^Bcl2^hi^ phenotype^36^. In our model, the most mature DP cells expressed higher amounts of CD44 and CD69, both of which increased from DP1 to DP2 to DP3; additionally, MHC class I expression increased significantly in DP3. Upon infection, the residual DP thymocytes expressed higher amounts of CD44 and MHC class I, indicating that the surviving population may be phenotypically more mature and/or activated (Fig. 2f–h). Upon infection, the intracellular amounts of anti-apoptotic Bcl2 are significantly elevated in the DP3 cells (Fig. 2i). Compared to the other two DP subsets, DP3 thymocytes are known to express higher amounts of Bcl2^5^ and it is possible that the DP3 cells that expressed higher amounts of Bcl2 survived better (Fig. 2i). This developmental stage dependent balance between pro- and anti-apoptotic molecules may be an underlying factor important for the resistance of DP3 cells and the susceptibility of DP1 and DP2 cells upon infection.

During the process of infection-induced thymic atrophy, SP cells have been reported to be largely unaffected^15^,^16^,^17^. However, in chronic infection studies using intracellular bacteria, all thymocyte subsets, including SP cells, were drastically reduced^18^,^19^. In this study, SP thymocytes remain largely unaltered but there is a modest reduction in the CD4^+^ SP compartment (Fig. 1d). Further phenotypic analysis demonstrated an accumulation of CD24^hi^CD3^hi^ cells in both CD4^+^ and CD8^+^ SP subsets (Figs 3a,b and 4a,b). Studying additional markers revealed that these CD24^hi/int^CD3^hi^ cells were less mature i.e., CD69^hi^CD62L^lo^ (Figs 3c–e and 4c–e) and, possibly, recently committed to the SP subset^33^. Accumulation of CD24^hi^ SP thymocytes during infection has not been reported earlier and further investigations are required to uncover the mechanisms involved. *Rag2* active cells in the thymus are functionally categorized on the basis of CD69 and MHC class I as semi-mature (CD69^+^ MHC class I^−^), mature 1 (CD69^+^ MHC class I^+^) and mature 2 (CD69^−^ MHC class I^+^)^37^. A prominent observation in our study was that the less mature CD24^int^ cells in control mice displayed the phenotype CD69^hi^MHC class I^lo^, whereas the more mature CD24^lo^ cells presented the opposite phenotype, CD69^lo^MHC class I^hi^. However, this pattern was altered during infection as MHC class I was upregulated in CD24^int^ cells (Figs 3g and 4g). It may be interesting to speculate that the processes that are at play during thymic atrophy are responsible for either modulating the maturation or selective survival of SP thymocyte subsets.

GCs are induced during *S*. Typhimurium infection and contribute to the death of immature thymocytes^17^. Homo-dimerization of the GC receptor followed by transactivation, but not trans-repression, is required for GC-induced thymocyte apoptosis^38^. Notably, RU486 binds to the GC receptor and reduces GC receptor-mediated transactivation^39^. Administration of the GC receptor antagonist RU486 to uninfected C57BL6 mice did not modulate the thymocyte subsets studied (Fig. S6). However, injection of RU486 post infection offered a modest protection from thymic atrophy Fig. 6a). Detailed analysis revealed that the reduction in cellularity of DN3 and DN4 cells was partially rescued, while the number of DN2 cells was fully recovered (Fig. 6d,e). Similar results were also obtained in another study where transgenic rats overexpressing a mutant GC receptor with increased ligand affinity were used^40^. Early lymphoid progenitors in mice and humans are highly sensitive to GCs^41^ and further studies are required to investigate the status of these progenitors in our model.

The involvement of GCs in depletion of DP thymocytes is well documented^42^,^43^,^44^. Overall, in the context of infection-induced thymic atrophy, RU486 mediated inhibition of GC signalling was more effective at rescuing DN and CD4^+^ SP cells compared to ISP and DP cells. This is counter-intuitive to published literature that suggests DP cells are most sensitive to GC-mediated apoptosis^15^,^21^. However, data regarding the expression of the GC receptor suggest that DN cells have the highest expression of the GC receptor followed by SP and then DP and ISP cells. Also, the GC antagonist RU486 does not affect expression of the GC receptor^44^,^45^. Therefore, further studies on the roles of the GC receptor are required during infection-induced thymic atrophy.

The pro-inflammatory cytokines Tnfα and Il6 increased with infection (Fig. S4e,f), which may lead to the progressive deterioration of the thymic architecture^35^, an important survival factor for developing thymocytes^46^. A significant part of our study was focussed on Ifnγ, an inflammatory cytokine, which has pleiotropic immunoregulatory effects on cells^47^. Although the infection burden is higher in mice lacking Ifnγ, the infection-linked thymic atrophy is lower^17^. The absence of Ifnγ reduced the overall degree of atrophy (Fig. 6a). DN and CD4^+^ SP cells were almost completely rescued from depletion, whereas the DP cells were moderately recovered (Fig. 6b,c). The most striking observation was that, the accumulation of DP3 thymocytes during infection was substantially reduced in *Ifnγ*^−/−^ mice (Fig. 7a,b). This observation may be interpreted as follows: Ifnγ protects DP3 cells during infection or the maturation of DP3 to SP cells may be slower due to high amounts of Ifnγ during infection. Alternately, a combination of both these possibilities may occur. DP2 cell numbers are reduced upon infection, thus restricting the availability of immature DP2 cells that can mature into the DP3 subset (Fig. 7a,b). Also, fewer CD24^hi^CD3^hi^ SP cells are accumulated in the *Ifnγ*^−/−^ mice as compared to wild type (Fig. 7c,d,e,f). In the context of infection, most likely, the DP3 cells in *Ifnγ*^−/−^ mice mature into CD8^+^ SP cells while not being replenished by DP2, hence resulting in a lower DP3 pool. The observation that Ifnγ induced upon infection inhibited the maturation of SP thymocytes is novel.

Three important aspects were observed in infected *Ifnγ*^−/−^ mice treated with RU486: First, ISPs were partially rescued, demonstrating the cooperative roles of both Ifnγ and GC in enhancing the depletion of ISPs. Second, RU486 treatment did not rescue the depletion of CD24^hi/int^CD3^hi^ SP cells in *Ifnγ*^−/−^ mice, indicating that Ifnγ produced during infection slowed the maturation and/or increased the survival of these thymocytes. Third, distinct differences were observed between the mature CD24^lo^CD4^+^ and CD24^lo^CD8^+^ SP thymocytes. Although CD24^lo^CD8^+^ SP were not affected during infection, the CD24^lo^CD4^+^ SP thymocyte numbers were reduced more in BL/6 compared to *Ifnγ*^−/−^ mice. Importantly, RU486 mediated rescue of these cells are more pronounced in BL/6 infected mice, revealing an important role of Ifnγ (Fig. 7).

Along with aging, numerous infections and stress conditions lead to a loss in thymic cellularity, which may subsequently lead to lymphopenia and loss in peripheral T cell receptor diversity. To circumvent this process, interventions are required which not only target pathogens but also boost the cellular immune system by reducing thymic atrophy^25^. Studies that lead to a better understanding of the subsets of thymocytes that are affected and the mechanisms that are involved in the differential susceptibility and maturation are important as they may lead to effective therapeutic interventions. Here, we have characterized changes that occur in different thymocyte subsets during an acute and lethal model of *Salmonella* infection in mice. By identifying these changes and studying the mechanisms involved, it may be possible to screen and select molecules which can ameliorate the extent of thymic atrophy in clinically relevant lymphopenic infections, e.g. acquired immunodeficiency syndrome. Molecules that rescue progenitor cells, such as DNs and ISPs, and enhance maturation of DPs to SPs may be better suited to reduce thymic depletion and repopulate the thymus, thereby maintaining optimal thymic output and enhancing the cellular host defence network during infections.

## Materials and Methods

### Bacterial cultures

*S*. Typhimurium NCTC 12023 strain was used for oral infections in mice^17^. An overnight pre-inoculum, cultured from a single bacterial colony grown on a *Salmonella*-*Shigella* agar plate was inoculated at 0.2% in 50 mL Luria broth and grown for 3–4 h (160 rpm, 37^◦^C) to achieve a log phase culture. The bacterial culture was resuspended in sterile PBS and mice were infected with ~10^8^ CFU in a total volume of 0.5 mL PBS by oral gavage^17^.

### Mice infections and inhibitors

BL/6.129S7-Ifng^tm1Ts^/J (*Ifnγ*^*−*/*−*^) mice were a generous gift from the National Institute of Immunology, New Delhi. C57BL/6 and *Ifnγ*^*−*/*−*^ mice were maintained and bred at the Central Animal Facility, IISc and 6–8 weeks old mice, of either sex, were used for experiments. All experimental groups contained a minimum of 3 to a maximum of 10 mice across different experiments. RU486 (Mifepristone), a progesterone and GC receptor antagonist, was dissolved in DMSO (Amresco Inc., USA) and injected intra-peritoneally in mice at 20 mg/kg dose, 12–16 h post-oral infection^17^. All reagents were purchased from Sigma –Aldrich Ltd. USA, unless stated otherwise.

### Ethics statement

All experiments were conducted in accordance with the Control and Supervision rules, 1998 of Ministry of Environment and Forests Act (Government of India) and Institutional Animal Ethics Committee, IISc. Mice were bred and maintained at the Central Animal Facility of IISc (Registration number: 48/1999/CPCSEA, dated 1/3/1999), approved by the Ministry of Environment and Forests, Government of India. Experimental protocols were approved by the ‘Committee for Purpose and Control and Supervision of Experiments on Animals’ (CPCSEA) and the permit number was CAF/Ethics/216/2011. Details of the national guidelines can be found on the website: http://envfor.nic.in/division/committee-purpose-control-and-supervision-experiments-animals-cpcsea.

### Mice Genotyping

The genotype of *Ifnγ*^*−*/*−*^ mice was confirmed by PCR. To obtain crude DNA, 2 mm tail samples were digested with 75 μL of 25 mM NaOH/0.2 mM EDTA solution at 95 °C for 1 h. The samples were incubated at room temperature for 15 min, followed by addition of 75 μL of 40 mM Tris HCl, pH 5.5. The tubes were centrifuged at 2000 g for 10 min and the supernatants were collected to perform the PCR. Genotyping was performed according to the protocol described on the Jackson laboratory website (https://www2.jax.org/protocolsdb/f?p=116:2:0::NO:2:P2_ MASTER_PROTOCOL_ID, P2_JRS_CODE:15019, 002287). Briefly, the PCR mix consisted of a common reverse primer (AGG GAA ACT GGG AGA GGA GAA ATA T), a forward primer specific for the mutant allele (CCT TCT ATC GCC TTC TTG ACG) and a forward primer specific for the wild type allele (AGA AGT AAG TGG AAG GGC CCA GAA G). The primers were obtained from Sigma- Aldrich Ltd. India. The amplified 500 bp mutant fragment and 210 bp wild type fragments were separated on a 2% agarose gel in TAE buffer.

### CFU analysis

On indicated days post infection, mice were euthanized using carbon dioxide and tissue samples/organs were collected in PBS. The organs were weighed and homogenized in 1 ml PBS and dilutions were plated on *Salmonella*-*Shigella* agar plates. CFU were enumerated after incubation at 37 °C for 12–16 h. Blood collected upon sacrificing the mice was kept at 4 °C to enable clotting and then centrifuged. The sera were stored at −20 °C for further analysis^17^.

### Isolation of lymph node cells and thymocytes

On indicated days post-infection, mice were sacrificed and the MLNs and thymi were collected in RPMI + 5% FBS (Gibco, USA). The tissues were washed in PBS, weighed and disrupted using a pair of forceps. The cell suspension was passed through a fine wire mesh to prepare a single cell suspension. Viable cell numbers were enumerated using Trypan blue exclusion assay with a haemocytometer^17^.

### Cytokines and cortisol measurement

Serum levels of Tnfα, Ifnγ and Il6 were quantified using ELISA kits (eBioscience, USA) and cortisol amounts were measured by an AccuBind ELISA kit (Monobind, Inc. USA) according to the manufacturer’s instructions^17^.

### Flow cytometric analysis

Analysis of cell surface markers and intracellular molecules was performed on FACSVerse flow cytometer, BD Biosciences USA. The list of antibodies and conjugates used for this study are listed in Supplementary Table 1. Each antibody was titrated prior to performing experiments using the appropriate dilutions. Briefly, cells were incubated with the indicated fluorochrome-conjugated antibodies specific to cell surface markers, at 4^◦^C for 45 min. Samples were washed and fixed with 0.5% paraformaldehyde before acquisition. For intracellular staining, the cells were stained with fluorochrome-conjugated antibodies, fixed using 4% PFA and resuspended in permeabilization buffer (HBSS + 0.2% saponin + 5% FBS + 0.01% sodium azide). Cells were incubated with specific antibodies against intracellular proteins, which were detected using appropriate fluorochrome tagged secondary antibodies. The samples were washed and fixed before acquiring on the flow cytometer. To set baseline instrument application settings and compensation settings for each fluorochrome measured, unstained and single fluorochrome stained cells were used. Preliminary experiments were conducted to obtain staining patterns for individual markers and these were compared to the results obtained by multi-colour flow cytometry^48^. Single cell populations were gated on the basis of forward scatter-area versus -height. This selection was subsequently applied to forward scatter area and side scatter area to gate on and analyze singlet populations of live lymphocytes.

CD4 and CD8 surface markers were used to gate on DP, DN and SP subsets. For further analysis of the DN population, cells were gated on the basis of CD44 and CD25 surface expression. For dissecting DP thymocyte subsets, CD5 and CD3 surface expression was analyzed and the surface or intracellular expression of various indicated markers was studied in DP1, DP2 and DP3 subsets. As Bax amounts in DP thymocytes were unchanged with infection, a positive control to study the expression of Bax in thymocytes treated with Dexamethasone (5 μM) was used (Fig. S5)^49^. To analyze SP populations, surface expression of CD24 and CD3 in the CD4^+^ or CD8^+^ SP subsets was examined. On the basis of CD24 expression, the cells were analyzed for expression of various indicated markers. FlowJo (9.8.5) and WinMDI software were used for flow cytometry data analysis. The data are represented as MFI (geometric mean fluorescence intensities) fold change, which was calculated by obtaining the MFI of the desired cell population and dividing the value with the MFI of uninfected thymocytes for the selected marker. The results have been pooled from multiple experiments performed on different days. The actual MFI values differ from experiment to experiment performed on different days. To reduce this variation, MFI fold change has been reported as it is more consistent and representative of the changes observed with multiple experiments.

### Statistical analysis

Data were plotted and analyzed using commercially available software (Microsoft Excel and GraphPad Prism 5). The quantified data from independent experiments are depicted as mean ± SEM. Statistically significant differences among various parameters were quantified by two-tailed Mann-Whitney test with 95% confidence interval. The p values were reported as follows: *p < 0.05, **p < 0.01, ***p < 0.001.

## Additional Information

**How to cite this article**: Majumdar, S. *et al*. Differential susceptibility and maturation of thymocyte subsets during *Salmonella* Typhimurium infection: insights on the roles of glucocorticoids and Interferon-gamma. *Sci. Rep.* **7**, 40793; doi: 10.1038/srep40793 (2017).

**Publisher's note:** Springer Nature remains neutral with regard to jurisdictional claims in published maps and institutional affiliations.

## Supplementary Material

Supplementary Information

## References

[b1] Yui M. A., Feng N. & Rothenberg E. V. Fine-scale staging of T cell lineage commitment in adult mouse thymus. J Immunol 185, 284–93, doi: 10.4049/jimmunol.1000679 (2010). 20543111 PMC4091773

[b2] Wilson A., Ewing T., Owens T., Scollay R. & Shortman K. T cell antigen receptor expression by subsets of Ly-2-L3T4- (CD8-CD4-) thymocytes. J Immunol 140, 1470–6 (1988). 2450128

[b3] Xiong J., Armato M. A. & Yankee T. M. Immature single-positive CD8^+^ thymocytes represent the transition from Notch-dependent to Notch-independent T-cell development. Int Immunol 23, 55–64, doi: 10.1093/intimm/dxq457 (2011). PMC3031305 21148236

[b4] Saini M., Sinclair C., Marshall D., Tolaini M., Sakaguchi S. & Seddon B. Regulation of Zap70 expression during thymocyte development enables temporal separation of CD4 and CD8 repertoire selection at different signaling thresholds. Sci Signal 3, ra23, doi: 10.1126/scisignal.2000702 (2010). 20332428

[b5] Sinclair C., Bains I., Yates A. J. & Seddon B. Asymmetric thymocyte death underlies the CD4:CD8 T-cell ratio in the adaptive immune system. Proc Natl Acad Sci USA 110, E2905–14, doi: 10.1073/pnas.1304859110 (2013). 23858460 PMC3732981

[b6] Yates A. J. Theories and quantification of thymic selection. Front Immunol 5, 13, doi: 10.3389/fimmu.2014.00013 (2014). 24550908 PMC3912788

[b7] Ladi E., Yin X., Chtanova T. & Robey E. A. Thymic microenvironments for T cell differentiation and selection. Nat Immunol 7, 338–43, doi: 10.1038/ni1323 (2006). 16550196

[b8] McCaughtry T. M., Wilken M. S. & Hogquist K. A. Thymic emigration revisited. J Exp Med 204, 2513–20, doi: 10.1084/jem.20070601 (2007). 17908937 PMC2118501

[b9] Aspinall R. Age-associated thymic atrophy in the mouse is due to a deficiency affecting rearrangement of the TCR during intrathymic T cell development. J Immunol 158, 3037–45 (1997). 9120255

[b10] Schilham M. W. . Critical involvement of Tcf-1 in expansion of thymocytes. J Immunol 161, 3984–91 (1998). 9780167

[b11] Savino W. The thymus is a common target organ in infectious diseases. PLoS Pathog 2, e62, doi: 10.1371/journal.ppat.0020062 (2006). 16846255 PMC1483230

[b12] Lynch H. E. . Thymic involution and immune reconstitution. Trends. Immunol 30, 366–73, doi: 10.1016/j.it.2009.04.003 (2009). 19540807 PMC2750859

[b13] Dooley J. & Liston A. Molecular control over thymic involution: from cytokines and microRNA to aging and adipose tissue. Eur J Immunol 42, 1073–9, doi: 10.1002/eji.201142305 (2012). 22539280

[b14] Fayad R. . Induction of thymocyte apoptosis by systemic administration of concanavalin A in mice: role of TNFα, IFNγ and glucocorticoids. Eur J Immunol 35, 2304–12, doi: 10.1002/eji.200526062 (2005). 16047339

[b15] Chen W. . Low dose aerosol infection of mice with virulent type A *Francisella tularensis* induces severe thymus atrophy and CD4^+^CD8^+^ thymocyte depletion. Microb Pathog 39, 189–96, doi: 10.1016/j.micpath.2005.08.005 (2005). 16257504 PMC1564440

[b16] Leite de Moraes M. C. . Studies on the thymus in Chagas’ disease. II. Thymocyte subset fluctuations in Trypanosoma cruzi-infected mice: relationship to stress. Scand J Immunol 33, 267–75, doi: 10.1111/j.1365-3083.1991.tb01772.x (1991). 1672774

[b17] Deobagkar-Lele M., Chacko S. K., Victor E. S., Kadthur J. C. & Nandi D. Interferon-γ- and glucocorticoid-mediated pathways synergize to enhance death of CD4(+) CD8(+) thymocytes during *Salmonella enterica* serovar Typhimurium infection. Immunology 138, 307–21, doi: 10.1111/imm.12047 (2013). 23186527 PMC3719942

[b18] Ross E. A. . Thymic function is maintained during Salmonella-induced atrophy and recovery. J Immunol 189, 4266–74, doi: 10.4049/jimmunol.1200070 (2012). 22993205 PMC3912538

[b19] Borges M. . Molecular and cellular mechanisms of Mycobacterium avium-induced thymic atrophy. J Immunol 189, 3600–8, doi: 10.4049/jimmunol.1201525 (2012). 22922815 PMC3593108

[b20] Liu B. . Severe influenza A(H1N1)pdm09 infection induces thymic atrophy through activating innate CD8(+)CD44(hi) T cells by upregulating IFN-γ. Cell Death Dis 5, e1440, doi: 10.1038/cddis.2014.323 (2014). 25275588 PMC4649502

[b21] Pérez A. R. . Thymus atrophy during *Trypanosoma cruzi* infection is caused by an immuno-endocrine imbalance. Brain Behav Immun 21, 890–900, doi: 10.1016/j.bbi.2007.02.004 (2007). 17412557

[b22] Koka P. S., Brooks D. G., Razai A., Kitchen C. M. & Zack J. A. HIV type 1 infection alters cytokine mRNA expression in thymus. AIDS. Res. Hum. Retroviruses 19, 1–12, doi: 10.1089/08892220360473916 (2003). 12581511

[b23] Sempowski G. D. . Leukemia inhibitory factor, oncostatin M, IL-6, and stem cell factor mRNA expression in human thymus increases with age and is associated with thymic atrophy. J Immunol 164, 2180–7, doi: 10.4049/jimmunol.164.4.2180 (2000). 10657672

[b24] Scofield V. L., Yan M., Kuang X., Kim S. J. & Wong P. K. The drug monosodium luminol (GVT) preserves crypt-villus epithelial organization and allows survival of intestinal T cells in mice infected with the ts1 retrovirus. Immunol Lett 122, 150–8, doi: 10.1016/j.imlet.2008.12.012 (2009). 19186189

[b25] Deobagkar-Lele M., Victor E. S. & Nandi D. c-Jun N-terminal Kinase is a critical node in the death of CD4^+^CD8^+^ thymocytes during *Salmonella enterica* serovar Typhimurium infection. Eur J Immunol 44, 137–49, doi: 10.1002/eji.201343506 (2014). 24105651

[b26] Farias-de-Oliveira D. A. . Caspase-8 and caspase-9 mediate thymocyte apoptosis in *Trypanosoma cruzi* acutely infected mice. J Leukoc Biol 93, 227–34, doi: 10.1189/jlb.1211589 (2013). 23159925

[b27] Wangdi T., Winter S. E. & Bäumler A. J. Typhoid fever: “you can’t hit what you can’t see”. Gut Microbes 3, 88–92, doi: 10.4161/gmic.18602 (2012). 22156762 PMC3370952

[b28] Zaki S. A. & Karande S. Multidrug-resistant typhoid fever: a review. J Infect Dev Ctries 5, 324–37, doi: 10.3855/jidc.1405 (2011). 21628808

[b29] Gordon M. A. Invasive nontyphoidal *Salmonella* disease: epidemiology, pathogenesis and diagnosis. Curr Opin Infect Dis 24, 484–9, doi: 10.1097/QCO.0b013e32834a9980 (2011). 21844803 PMC3277940

[b30] Kubo R. T., Born W., Kappler J. W., Marrack P. & Pigeon M. Characterization of a monoclonal antibody which detects all murine alpha beta T cell receptors. J Immunol 142, 2736–42 (1989). 2467936

[b31] Levelt C. N., Carsetti R. & Eichmann K. Regulation of thymocyte development through CD3. II. Expression of T cell receptor beta CD3 epsilon and maturation to the CD4+8+ stage are highly correlated in individual thymocytes. J Exp Med 178, 1867–1875 (1993). 7504052 10.1084/jem.178.6.1867 PMC2191302

[b32] Xu X. . Maturation and emigration of single-positive thymocytes. Clin Dev Immunol 282870, doi: 10.1155/2013/282870 (2013). 24187562 PMC3804360

[b33] Fredin F. . Dextran sulfate sodium-induced colitis generates a transient thymic involution–impact on thymocyte subsets. Scand J Immunol 65, 421–9, doi: 10.1111/j.1365-3083.2007.01923.x (2007). 17444952

[b34] Hyland L., Villarreal-Ramos B., Clarke B., Baaten B. & Hou S. Bone marrow immunosuppression in *Salmonella*-infected mice is prolonged following influenza virus infection. Exp Hematol 33, 1477–85, doi: 10.1016/j.exphem.2005.09.005 (2005). 16338490

[b35] Liepinsh D. J. . Accelerated thymic atrophy as a result of elevated homeostatic expression of the genes encoded by the TNF/lymphotoxin cytokine locus. Eur J Immunol 39, 2906–15, doi: 10.1002/eji.200839191 (2009). 19735075 PMC13404808

[b36] Punt J. A., Suzuki H., Granger L. G., Sharrow S. O. & Singer A. Lineage commitment in the thymus: only the most differentiated (TCRhibcl-2hi) subset of CD4^+^CD8^+^ thymocytes has selectively terminated CD4 or CD8 synthesis. J Exp Med 184, 2091–9, doi: 10.1084/jem.184.6.2091 (1996). 8976166 PMC2196385

[b37] Xing Y., Wang X., Jameson S. C. & Hogquist K. A. Late stages of T cell maturation in the thymus involve NF-κB and tonic type I interferon signaling. Nat Immunol 17, 565–73, doi: 10.1038/ni.3419 (2016). 27043411 PMC4837029

[b38] Reichardt H. M. . DNA binding of the glucocorticoid receptor is not essential for survival. Cell 93, 531–41 (1998). 9604929 10.1016/s0092-8674(00)81183-6

[b39] Heck S. . A distinct modulating domain in glucocorticoid receptor monomers in the repression of activity of the transcription factor AP-1. EMBO J 13, 4087–4095 (1994). 8076604 10.1002/j.1460-2075.1994.tb06726.x PMC395330

[b40] van den Brandt J. . Enhanced glucocorticoid receptor signaling in T cells impacts thymocyte apoptosis and adaptive immune responses. Am J Pathol 170, 1041–53, doi: 10.2353/ajpath.2007.060804 (2007). 17322387 PMC1864890

[b41] Igarashi H. . Early lymphoid progenitors in mouse and man are highly sensitive to glucocorticoids. Int Immunol 17, 501–11, doi: 10.1093/intimm/dxh230 (2005). 15746243

[b42] Talabér G. . Mitochondrial translocation of the glucocorticoid receptor in double-positive thymocytes correlates with their sensitivity to glucocorticoid-induced apoptosis. Int Immunol 21, 1269–76, doi: 10.1093/intimm/dxp093 (2009). 19737783

[b43] Ryan J. A., Brunelle J. K. & Letai A. Heightened mitochondrial priming is the basis for apoptotic hypersensitivity of CD4+ CD8+ thymocytes. Proc Natl Acad Sci USA 107, 12895–900, doi: 10.1073/pnas.0914878107 (2010). 20615979 PMC2919900

[b44] Brewer J. A., Sleckman B. P., Swat W. & Muglia L. J. Green fluorescent protein-glucocorticoid receptor knockin mice reveal dynamic receptor modulation during thymocyte development. J Immunol 169, 1309–18, doi: 10.4049/jimmunol.169.3.1309 (2002). 12133953

[b45] Boldizsár F. . Low glucocorticoid receptor (GR), high Dig2 and low Bcl-2 expression in double positive thymocytes of BALB/c mice indicates their endogenous glucocorticoid hormone exposure. Immunobiology 211, 785–96, doi: 10.1016/j.imbio.2006.06.005 (2006). 17113916

[b46] Mohtashami M. & Zúñiga-Pflücker J. C. Three-dimensional architecture of the thymus is required to maintain delta-like expression necessary for inducing T cell development. J Immunol 176, 730–4, doi: 10.4049/jimmunol.176.2.730 (2006). 16393955

[b47] Saha B., Jyothi Prasanna S., Chandrasekar B. & Nandi D. Gene modulation and immunoregulatory roles of interferon gamma. Cytokine 50, 1–14, doi: 10.1016/j.cyto.2009.11.021 (2010). 20036577

[b48] Tan C. . Ten-color flow cytometry reveals distinct patterns of expression of CD124 and CD126 by developing thymocytes. BMC Immunol 12, 36, doi: 10.1186/1471-2172-12-36 (2011). 21689450 PMC3130696

[b49] Hoijman E., Rocha Viegas L., Keller Sarmiento M. I., Rosenstein R. E. & Pecci A. Involvement of Bax protein in the prevention of glucocorticoid-induced thymocytes apoptosis by melatonin. Endocrinology 145, 418–25, doi: 10.1210/en.2003-0764 (2004). 14500572

