# Supplementary material for: Differential susceptibility and maturation of thymocyte subsets during Salmonella Typhimurium infection: insights on the roles of glucocorticoids and Interferon-gamma
Source: Sci Rep. 2017 Jan 16;7:40793. doi: 10.1038/srep40793 (PMC5238503; doi:10.1038/srep40793)
Supplement: Supplementary Information [file srep40793-s1.pdf]

Differential susceptibility and maturation of thymocyte subsets during *Salmonella* Typhimurium infection: insights on the roles of glucocorticoids and Interferon-gamma

Shamik Majumdar[1], Mukta Deobagkar-Lele[1], Vasista Adiga[2,3], Abinaya Raghavan[1], Nitin Wadhwa[1], Syed Moiz Ahmed[1], Supriya Rajendra Rananaware[1], Subhashish Chakraborty[1], Omana Joy[3] and Dipankar Nandi[1,2,3,*]

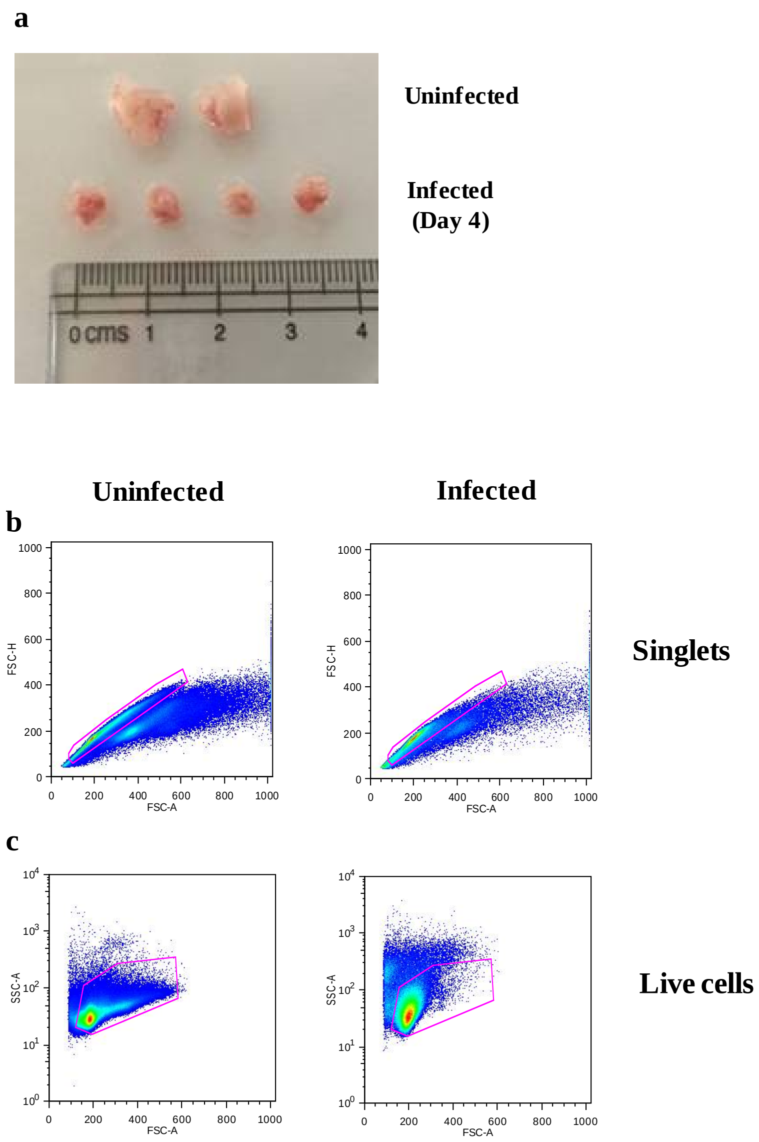

Figure S1: Gating strategy for analysis of live single cells. C57BL/6 mice were infected orally with *S*. Typhimurium and sacrificed on day 4 post infection. Representative image shows the sizes of dissected (**a**) thymi from uninfected and infected mice. Thymocytes were stained and data was acquired on the flow cytometer. Gating strategy to identify (**b**) singlet populations of thymocytes is shown. These were further gated on the basis of side scatter-area versus forward scatter-area to distinguish (**c**) single live thymocytes. For demonstration of gatings, data are shown from one experiment representative of at least three independent experiments.

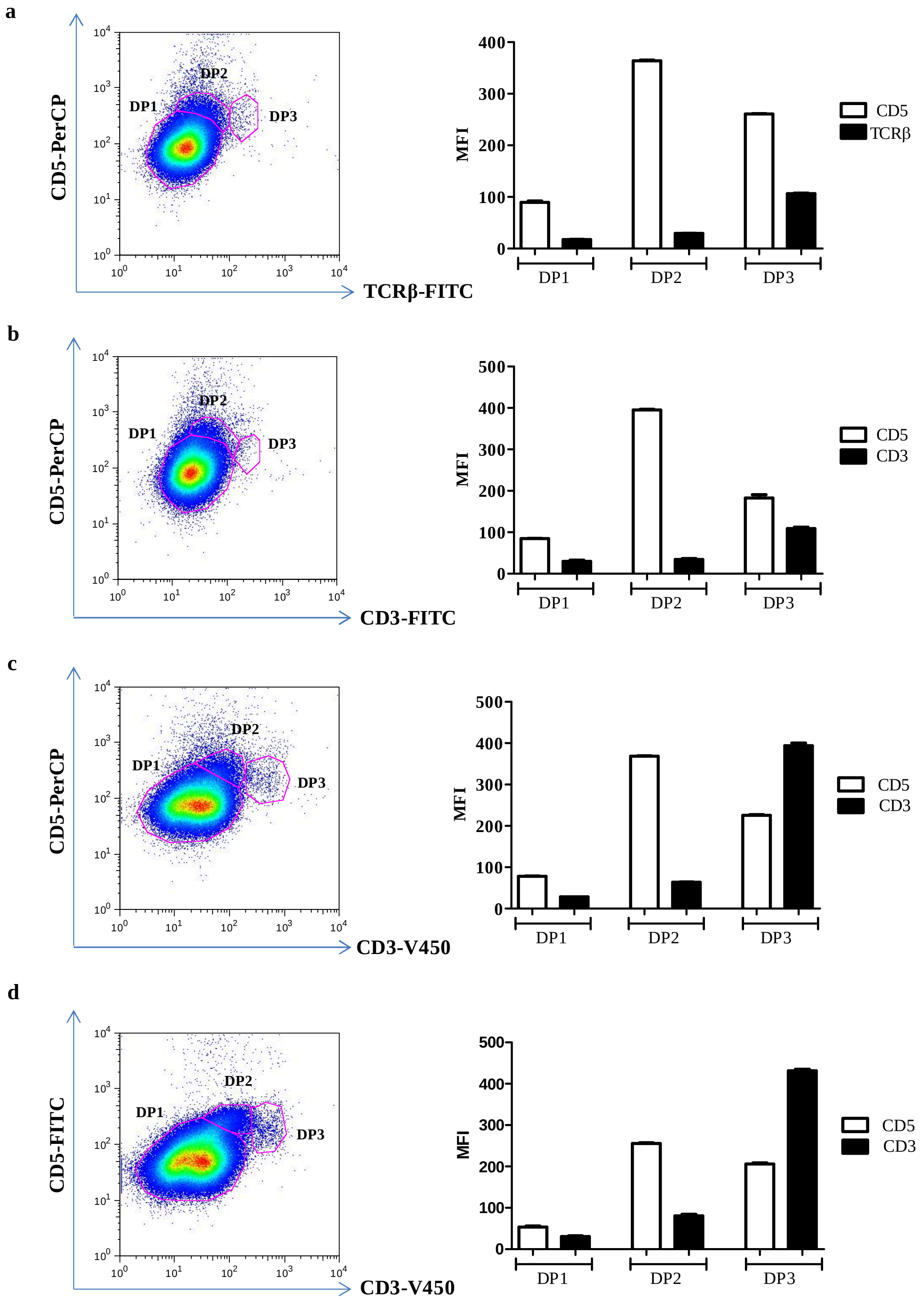

Figure S2: Comparison of CD5 and TCRβ or CD3 staining patterns using specific antibodies conjugated to different fluorescent tags. Uninfected C57BL/6 mice were sacrificed and the thymi were collected. Thymocytes were stained for cell surface expression of (**a**) CD5 versus TCRβ or (**b**,**c**,**d**) CD5 versus CD3 using antibodies conjugated to the mentioned fluorescent tags.

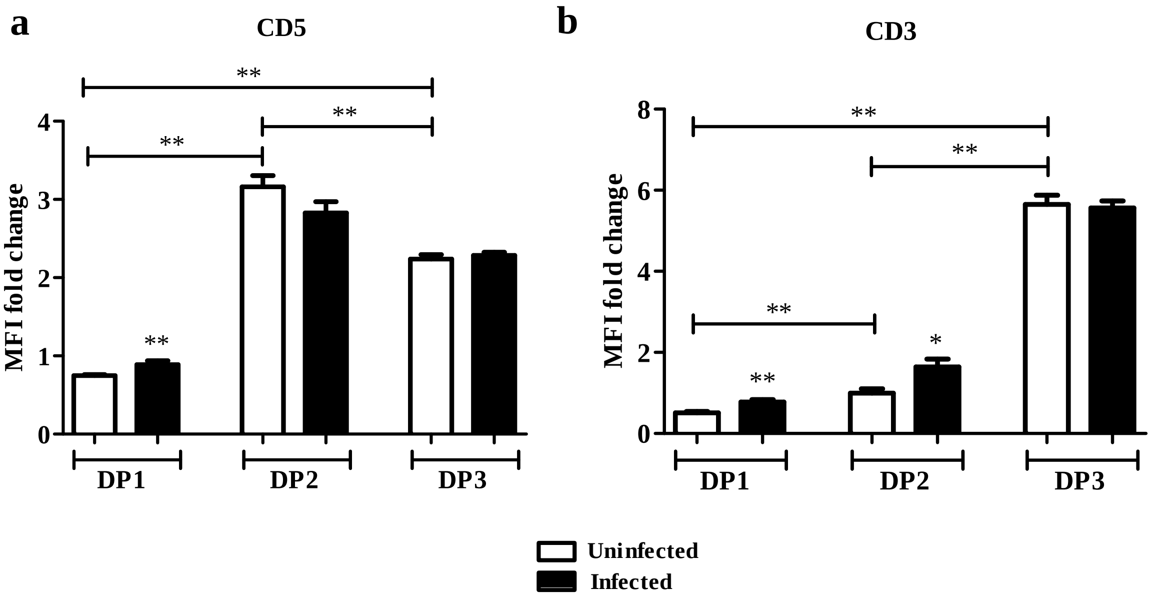

Figure S3: Classification of DP cells into DP1, DP2 and DP3 cells. Thymocytes from uninfected and infected mice were stained for cell surface expression of CD4, CD8, CD5 and CD3 and analyzed on the flow cytometer. The CD4 and CD8 DP cells were gated to categorize DP on the basis of (**a**) CD5 and (**b**) CD3 as DP1 being $CD5^{lo}CD3^{lo}$, DP2 being $CD5^{hi}CD3^{int}$ and DP3 being $CD5^{int}CD3^{lo}$. In subsequent experiments, these gates were applied throughout to identify DP subsets. Data are shown as mean ± SEM of six mice per group. *$p \leq 0.05$, **$p \leq 0.01$, and ***$p \leq 0.001$, two-tailed Mann-Whitney test.

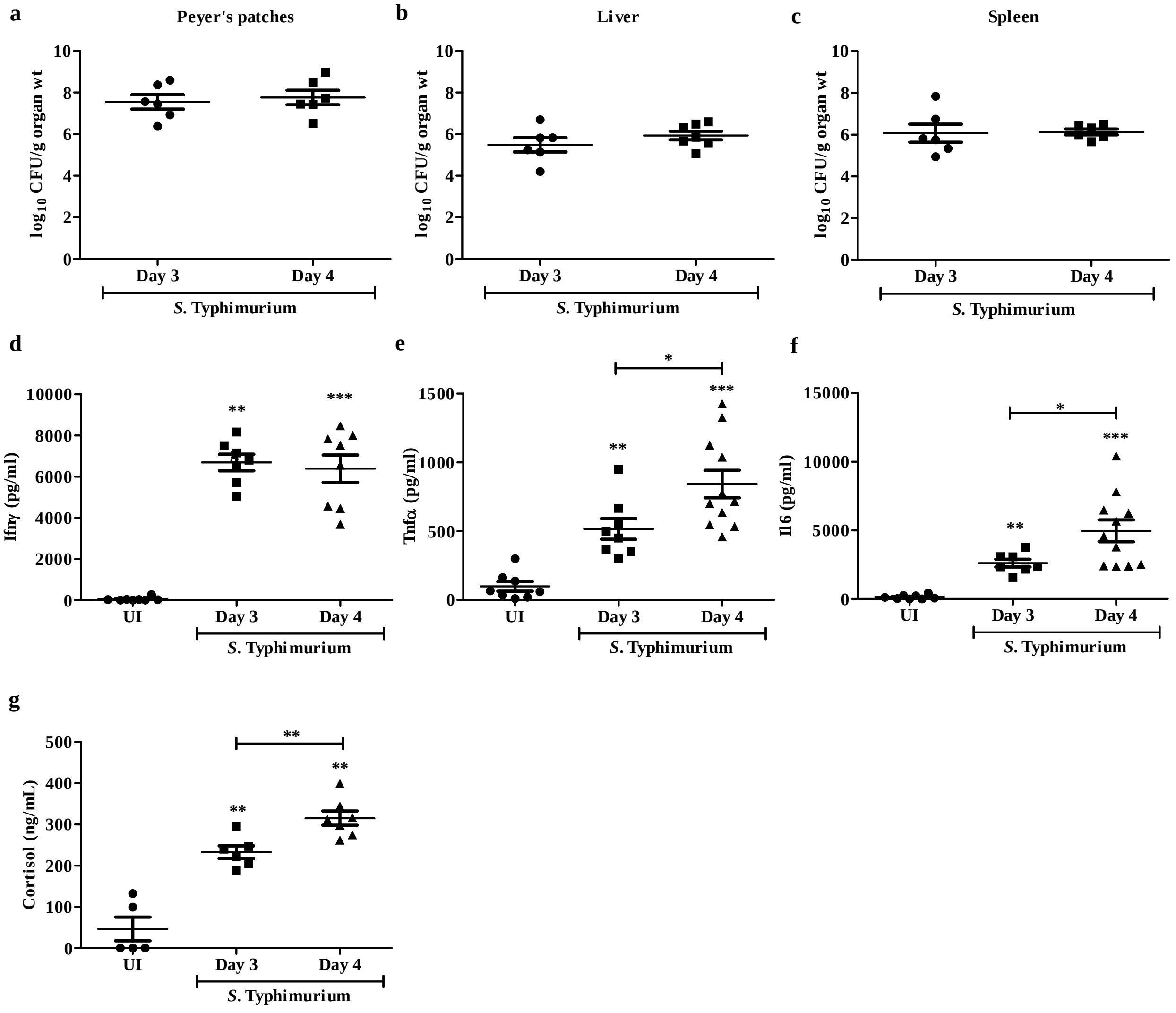

Figure S4: Tnfα, Il6 and cortisol amounts in sera are lower in infected mice displaying partial thymic atrophy. Uninfected (UI) control mice along with infected mice with partial (day 3) or acute (day 4) thymic atrophy were sacrificed and the organs and sera were collected. The bacterial CFU in (**a**) Peyer's patches, (**b**) liver and (**c**) spleen were calculated. The (**d**) Ifnγ, (**e**) Tnfα, (**f**) Il6 and (**g**) cortisol amounts in sera were also quantified. Data are shown as mean ± SEM of six to nine mice per group. *$p \leq 0.05$, , and ***$p \leq 0.001$, two-tailed Mann-Whitney test.

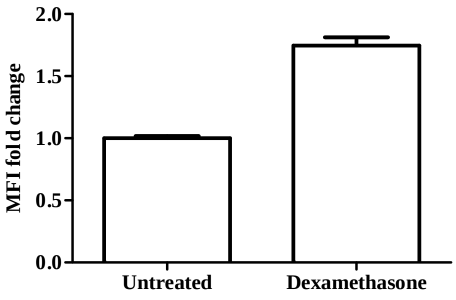

Figure S5: Dexamethasone used as positive control for quantification of intracellular Bax amounts. Thymocytes from uninfected C57BL/6 mouse were treated with dexamethasone for 4 hours. The cells were washed and stained for cell surface expression of CD4 and CD8 and intracellular Bax amounts. Bax amounts were measured in DP thymocytes.

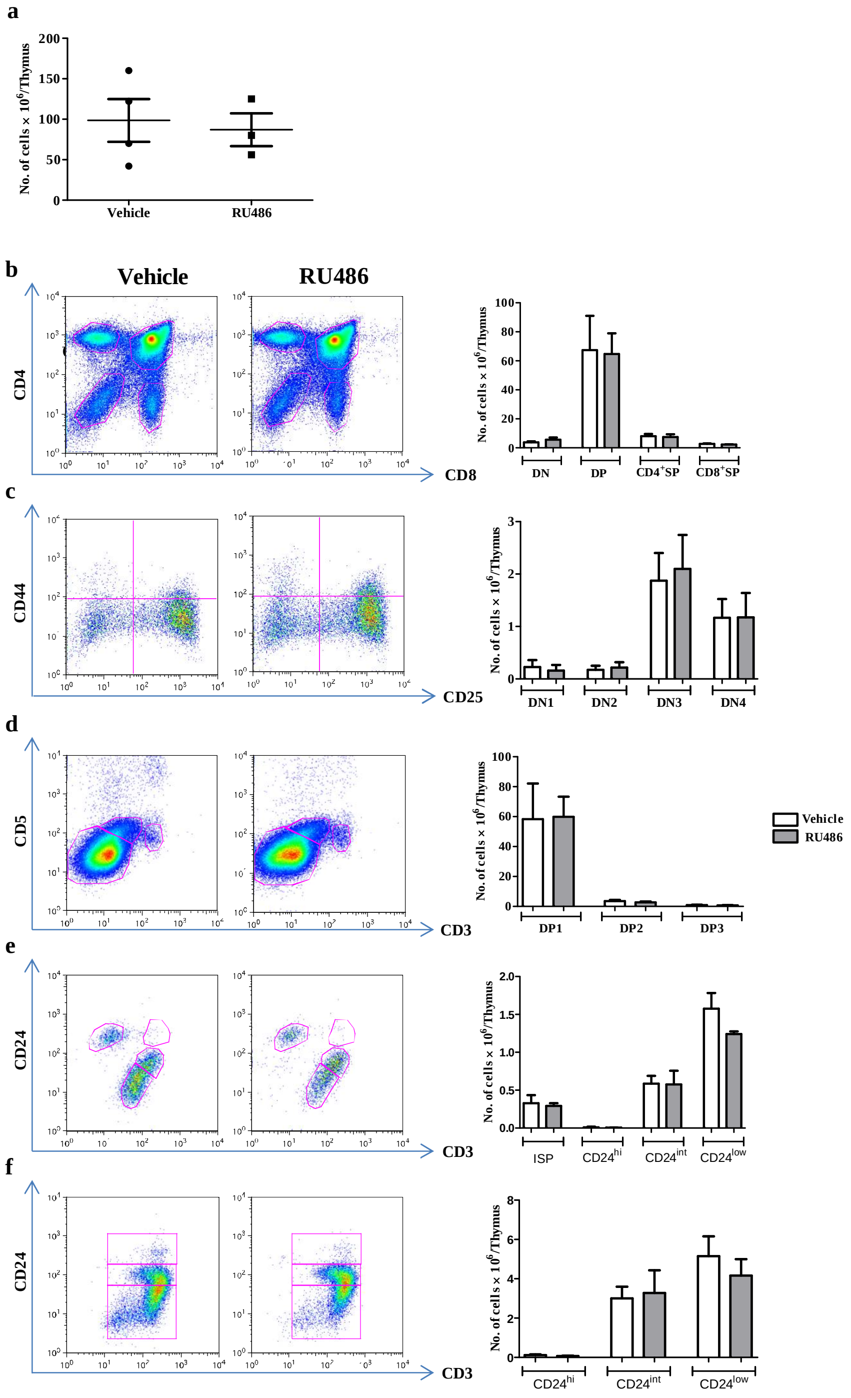

Fig S6: Thymocyte subpopulations are not affected by R486 treatment in uninfected mice. C57BL/6 mice were treated intra peritoneally with 20mg/kg of RU486. Three days post administration, RU486 along with vehicle treated mice were sacrificed and (**a**) the total number of live thymocytes were quantified. Cells from different thymocyte subsets were analyzed to quantify the cell numbers in the following populations: (**b**) total DN, DP, $CD4^+$ SP and $CD8^+$ SP subsets. (**c**) DN thymocytes, (**d**) DP cells, (**e**) $CD8^+$ SP and (**f**) $CD4^+$ SP subsets. Data are shown as mean ± SEM of three to four mice per group. No differences in thymic sub populations were observed in the two groups studied using the two-tailed Mann-Whitney test.

Antigen	Clone	Catalog number	Conjugate	Supplier	Titre used for staining
CD3e	17A2	47-0032-82	APC-eFlour®780	eBioscience	1:800
CD3e	500A2	560801	V450	BD	1:200
CD3e	145-2C11	11-0031-85	FITC	eBioscience	1:200
CD4	RM4-5	553051	APC	BD	1:800
CD4	GK1.5	100422	PE/Cy7	BioLegend	1:2000
CD4	RM4-5	48-0042-82	eFluor®450	eBioscience	1:200
CD5	53-7.3	553021	FITC	BD	1:500
CD5	53-7.3	13-0051-85	Biotin	eBioscience	1:100
CD8a	53-6.7	12-0081-85	PE	eBioscience	1:600
CD8a	53-6.7	100710	PE/Cy5	BioLegend	1:2000
CD8a	53-6.7	130-102-814	PE-Vio770	Miltenyi Biotec	1:200
CD24	M1/69	563115	BV510	BD	1:400
CD25	PC61.5	12-0251-82	PE	eBioscience	1:600
CD44	IM7	14-0441-85	Purified	eBioscience	1:200
CD44	IM7	45-0441-82	PerCP-Cyanine5.5	eBioscience	1:400
CD62L	MEL-14	562910	BV421	BD	1:5000
CD69	H1.2F3	25-0691-82	PE/Cy7	eBioscience	1:3000
MHCI	34-1-2S	46-5998-82	PerCP-eFlour®710	eBioscience	1:600
TCRβ	H57-597	11-5961-81	FITC	eBioscience	1:400
Bax	6A7	556467	Purified	BD	1:200
Bcl2	3F11	554218	Purified	BD	1:200
Streptavidin	-	554064	PerCP	BD	1:50
Goat anti-Rat IgG	Polyclonal	112-095-062	FITC	Jackson	1:200
Goat anti-Armenian Hamster IgG	Polyclonal	127-115-160	PE	Jackson	1:200
Goat anti-Mouse IgG	Polyclonal	115-095-020	FITC	Jackson	1:200
Goat anti-Mouse IgG	Polyclonal	115-115-164	PE	Jackson	1:200

Table S1: List of antibodies and their conjugates used in this study. Cell surface staining were performed using antibody dilutions made in PBS supplemented with 5% FCS. For intracellular staining, the antibodies were diluted in permeabilization buffer (HBSS containing 0.2% saponin, 5% FCS and 0.01% sodium azide). Secondary antibodies were used depending on the primary antibodies used.
